# Supplementary material for: Freeze-like responses to pain in humans and its modulation by social context
Source: PeerJ. 2020 Nov 19;8:e10094. doi: 10.7717/peerj.10094 (PMC7680627; doi:10.7717/peerj.10094)
Supplement: Supplemental Information 3 [file peerj-08-10094-s003.docx]

**Supplementary material 1**

**Methods**

In addition to these 40 stimuli five sets of morphed stimuli were created using Fantamorph 5 (Abrosoft, Beijing, China). The angry and happy facial stimuli of any one model were morphed at different degrees of emotional intensity (at 17%, 33%, 50%, 67% and 83%), resulting in an additional 100 stimuli (see Supplementary Figure 1).

**Generalization phase.** The five generalization blocks (B7-11) had the same structure as the experimental blocks. However, instead of a threatening and safe context, morphed variations of the two facial expressions were used with varying degrees of emotional intensity (17%, 33%, 50%, 67%, 83%) resulting in five generalization contexts (G1-5, see Supplementary Figure 1).

**Statistical analyses.** A 7 [Context (threat, G1-5, safe)] x 2 [Stimulus type (CS+/context-alone)] RM ANOVAs was run to investigate the generalization of social context effects on pain-US expectancy ratings. Separate RM ANOVAs with Context (threat, G1-5, safe) as factor and centered STAI-T scores as covariate were run to investigate potential differences in the contextual gradient with regard to pain expression, heart rate, pain intensity and unpleasantness ratings. Note that we only included the last threat and safe block in these analyses, since these blocks mark the end of acquisition where learning was maximal. Because of drop-out, only 32 participants were included for these analyses.

**Results**

**Manipulation check.** As anticipated, morphed faces were rated as less pleasant, linear trend, *F*(1, 31) = 27.63, *p* < .01, $\eta_{p}^{2}$ = .47, and more threatening, linear trend, *F*(1, 31) = 26.34, *p* < .01, $\eta_{p}^{2}$ = .46, the more they resembled the original angry facial stimuli. Furthermore, participants indicated that they felt increasingly unhappy, linear trend, *F*(1, 31) = 13.05, *p* < .01, $\eta_{p}^{2}$ = .30, but not more aroused, linear trend, *F*(1, 31) = 1.50, *p* = .23, $\eta_{p}^{2}$ = .05, or less in control, *F*(1, 31) < 1, *p* = .54, $\eta_{p}^{2}$ = .01, as the faces resembled the angry facial stimuli (see Supplementary Figure 2).

**Pain-US expectancy.** Social context did not affect the differentiation between CS+ and context-alone trials, Context x Stimulus type, *F*(6, 25) = 1.64, *p* = .18, $\eta_{p}^{2}$ = .28. However, as expected, participants still indicated higher pain-US expectancy in CS+ trials compared to context-alone trials, Stimulus, *F*(1,30) = 186.47, *p* < .01, $\eta_{p}^{2}$ = .86, (see Supplementary Figure 3).

**Pain intensity and unpleasantness.** Social context did not modulate pain intensity ratings, Context, *F* < 1, *p* = .91, $\eta_{p}^{2}$ = .08, or pain unpleasantness ratings, Context, *F* < 1, *p* = .97, $\eta_{p}^{2}$ = .05 (see Supplementary Figure 3).

**Heart rate.** There was no interaction between social context and trait anxiety, *F*(6, 24) < 3, *p* = .59, $\eta_{p}^{2}$ = .16, or social context itself on heart rate, *F*(6, 24) = 1.37, *p* = .27, $\eta_{p}^{2}$ = .26.

**Pain expression.** There was no interaction between social context and trait anxiety, *F*(6, 25) < 1, *p* = .65, $\eta_{p}^{2}$ = .14, and social context itself did not modulate pain expression, *F*(6, 25) < 1, *p* = .68, $\eta_{p}^{2}$ = .08 (see Supplementary Figure 3).

**Body sway.** One participant was identified as outlier because of excessive movements, as reflected in Z-scores greater than 4 on the body sway measures^[[1]](#footnote-1)^. Consequently, we decided to remove this participant from the analyses. The three-way interaction was not significant, Context x Stimulus type x Trait anxiety, *F*(6,23) = 1.23, *p* = .33, $\eta_{p}^{2}$ = .24. However, as expected there was less body sway in trials with the auditory stimulus, *M*_CS+_ = .18, *SD*_CS+_ < .05, compared to trials without it, *M*_context-alone_ = .17, *SD*_context-alone_ = .05, Stimulus type, *F*(1,28) = 9.49, *p* < .01, $\eta_{p}^{2}$ = .25 (see Supplementary Figure 3).

1. The results of these analyses remained the same even when the outliers were included. [↑](#footnote-ref-1)
